# Comparative pathology boards facilitate the translation of knowledge between canine and human cancer patients

**DOI:** 10.1111/bpa.70013

**Published:** 2025-05-05

**Authors:** Jessica A. Beck, Christina Mazcko, Sara Belluco, Mireille Bitar, Daniel Brat, Jonathan W. Bush, Rati Chkheidze, Kara N. Corps, Chad Frank, Caterina Giannini, Craig Horbinski, Jason T. Huse, Jennifer W. Koehler, Andrew D. Miller, C. Ryan Miller, M. Gerard O'Sullivan, Joanna J. Phillips, Daniel R. Rissi, Courtney R. Schott, Anat Stemmer‐Rachamimov, Stephen Yip, Amy K. LeBlanc

**Affiliations:** ^1^ Comparative Oncology Program National Cancer Institute, National Institutes of Health Bethesda Maryland USA; ^2^ Université de Lyon, VetAgro Sup, UPSP 2021.A104 ICE ‘Interactions Cellules Environnement’ Axe Cancérologie Lyon France; ^3^ Department of Pathology University of Texas Southwestern Medical Center Dallas Texas USA; ^4^ Department of Pathology Feinberg School of Medicine, Northwestern University Chicago Illinois USA; ^5^ Department of Pathology and Laboratory Medicine BC Children's Hospital, University of British Columbia Vancouver British Columbia Canada; ^6^ Department of Pathology UAB Heersink School of Medicine, University of Alabama at Birmingham Birmingham Alabama USA; ^7^ Department of Veterinary Biosciences College of Veterinary Medicine, The Ohio State University Columbus Ohio USA; ^8^ Department of Microbiology, Immunology and Pathology Colorado State University Fort Collins Colorado USA; ^9^ Laboratory Medicine and Pathology Mayo Clinic Comprehensive Cancer Center Rochester New York USA; ^10^ Department of Pathology University of Texas MD Anderson Cancer Center Houston Texas USA; ^11^ Department of Translational Molecular Pathology University of Texas MD Anderson Cancer Center Houston Texas USA; ^12^ Department of Pathobiology Auburn University College of Veterinary Medicine Auburn Alabama USA; ^13^ Department of Population Medicine and Diagnostic Sciences College of Veterinary Medicine, Cornell University Ithaca New York USA; ^14^ Department of Veterinary Population Medicine College of Veterinary Medicine, University of Minnesota St. Paul Minnesota USA; ^15^ Department of Neurological Surgery University of California San Francisco San Francisco California USA; ^16^ Department of Pathology University of California San Francisco San Francisco California USA; ^17^ Athens Veterinary Diagnostic Laboratory, Department of Pathology College of Veterinary Medicine, University of Georgia Athens Georgia USA; ^18^ Department of Pathobiology Ontario Veterinary College, University of Guelph Guelph Ontario Canada; ^19^ Department of Pathology Massachusetts General Hospital, Harvard Medical School Boston Massachusetts USA

**Keywords:** animal disease models, comparative oncology, glioma, histology, meningioma, pathology

## Abstract

Comparative pathology boards bring together anatomic pathologists with expertise in canine and human histology to identify shared features, including immune or TME components, tumor subtypes, or prognostic tissue biomarkers. This article summarizes feedback to improve future initiatives and enhance the translational relevance of comparative oncology for human patients.

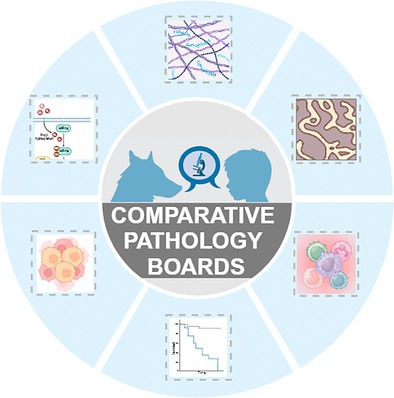

Comparative pathology boards (CPBs) bring together anatomic pathologists with expertise in canine (DVM) and human (MD) pathology to perform parallel review of canine tissue histology. CPB objectives can include characterization of tumor subtypes or identification of tissue‐based biomarkers with potential relevance for human patients (Figure S1). The National Cancer Institute's Comparative Oncology Program (COP) has supported three CPBs. These efforts originated within the Comparative Brain Tumor Consortium, which aimed to establish the relevance of the tumor‐bearing pet dog in human neuro‐oncology research [1]. With the goal of improving future CPB initiatives, an online survey of COP CPB pathologists was conducted using Research Electronic Data Capture [2]. Twenty‐one responses were received from the glioma (*n* = 10; seven DVM, three MD), meningioma (*n* = 9; six DVM, three MD), and osteosarcoma CPBs (*n* = 2; one MD, one DVM; Figure 1A). Because the study conditions varied, results are independently reported by CPB. Four veterinary pathologists with neuropathology expertise participated in both the glioma and meningioma CPBs. No MD pathologists were on more than one CPB. Responses from the osteosarcoma CPB are included within the text but omitted from graphs.

**FIGURE 1:**
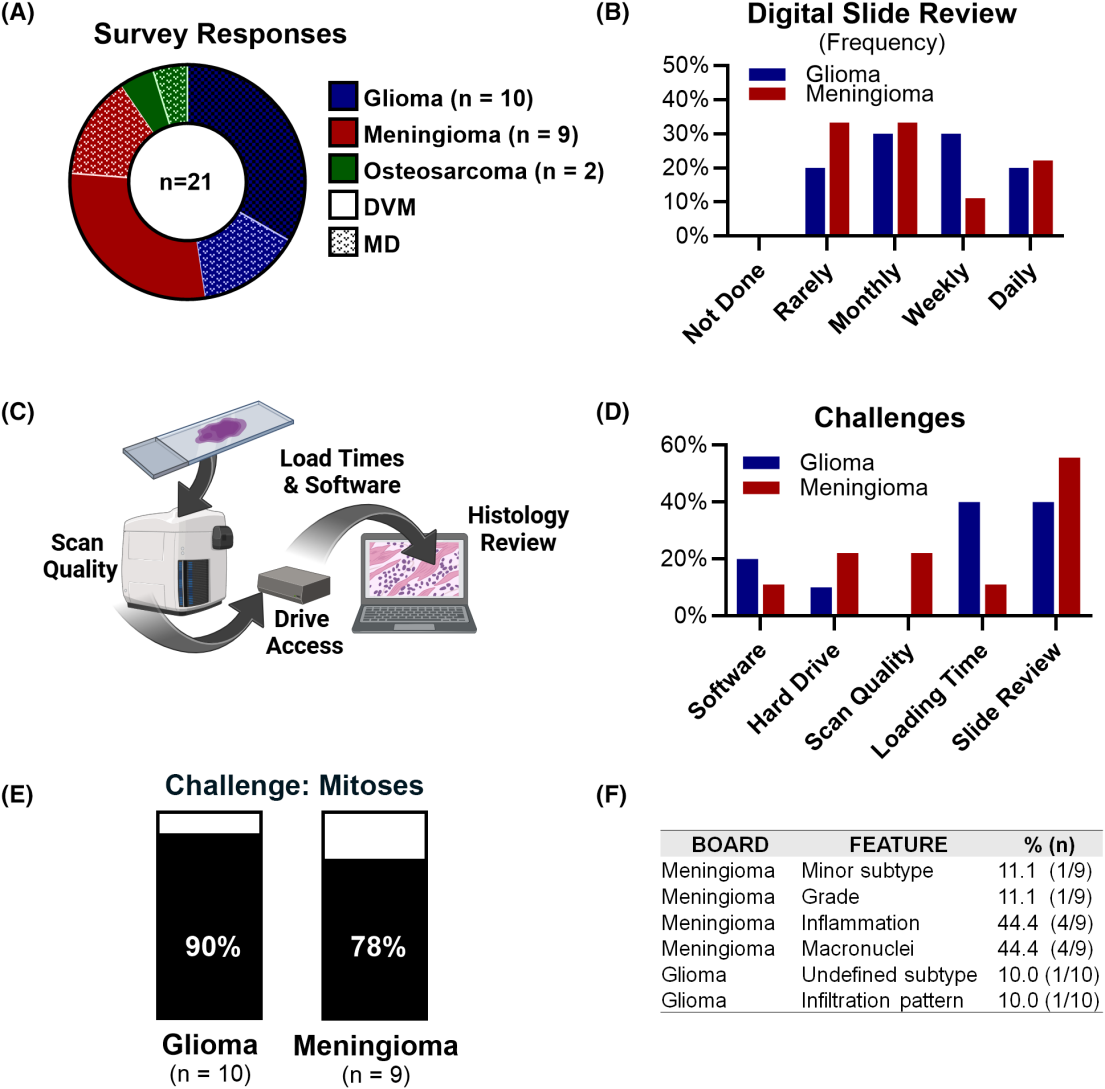
Review challenges for comparative pathology boards (CPBs). (A) Number and specialty (DVM, MD) of survey respondents from the glioma, meningioma, and osteosarcoma CPBs. (B) Frequency of digital slide review prior to beginning CPB activities. (C) General CPB workflow of slide scanning and review. (D) Percent of survey respondents that experienced each review challenge. (E) Percent of survey respondents that reported difficulty in identifying mitotic figures in digital slides. (F) Percent (and *n*) of survey respondents that reported additional features as difficult to assess during digital slide review.

All survey respondents agreed or strongly agreed that the mixed composition (MD + DVM) was a strength of the COP CPB process. Although human grading systems have been applied to multiple canine cancer types including meningioma [3], input from both sides allows researchers to better understand how canine cancers mimic or differ from their human counterparts. For example, MD‐trained pathologists on the glioma CPB were significantly less likely to report infiltration and necrosis or to diagnose oligodendrogliomas compared to DVM‐trained pathologists [4]. This may be reflective of how histologic features present in canine gliomas or differences in tumor type caseload across professions. Furthermore, MD‐trained pathologists routinely incorporate molecular data in their diagnosis, which is not a standard component of the current veterinary workflow. Identifying and understanding these differences is key to defining the value of the tumor‐bearing pet dog in comparative research.

Engaging pathologists with varied backgrounds provides different perspectives to the CPB. Most respondents described their career focus as a mixture of diagnostic pathology and applied research (Figure S2). The glioma CPB included both board‐certified pathologists and pathology residents engaged in PhD doctoral research [4]. Building a solid backbone with strong subject matter expertise is critical for CPB success and for buy‐in following publication. In addition, CPB pathologists must confidently share their opinions on a tumor, which can be difficult for some, such as early career pathologists. Conversely, early career pathologists can bring a new perspective and may be more open to reevaluating their approach.

Review of whole‐slide images over traditional glass slides streamlines the CPB process. All CPB members reported using digital slides previously, but most reviewed them infrequently (Figure 1B), highlighting the importance of software training sessions. Slow loading time was a common challenge (Figure 1C,D). Storing scans on a publicly available database rather than using hard drives should be considered, although it would be important to have a method for pathologists to annotate tissues without biasing others. During the review, mitoses were considered the most difficult feature to assess (Figure 1E). Other features reported as difficult to evaluate included inflammation and macronuclei (meningioma CPB; Figure 1F). Access to high‐resolution monitors may help. Literature review can also be invaluable; for example, mitotic figure references may help improve confidence and interpretation in digital slides [5]. Histologic features with higher subjectivity are particularly problematic, underscoring the importance of iterative case review and pre‐review meetings to refine morphologic criteria, increase interobserver agreement, and improve reproducibility of grading systems.

Clear messaging regarding the time commitment for CPB participation is critical for member recruitment and retention. Although reviewing different features, most CPB members reported spending 5–15 min per case (Figure 2A). This gives future CPBs a meaningful benchmark by which to request pathologists' participation. Considering that CPBs may review 100–200 cases, the review alone can take up to 50 h. This is in addition to pre‐review training, post‐review discussion, and publication efforts. Members of the glioma CPB, which has already published their findings [1, 4], estimated a total of >60 h (Figure 2B). However, few pathologists reported receiving time off service to support CPB activities (Figure 2C). Because the CPB requires a substantial time commitment and perseverance, it is important to consider individuals with a proven track record of productive collaborative work and an ability to complete projects in a timely manner. This is compounded by the need to complete reviews efficiently to prevent diagnostic drift. Taken together, time constraints remain a critical challenge. Methods to reduce this burden moving forward should be considered, including the use of digital pathology analyses and AI algorithms which can be developed based on consensus CPB annotations.

**FIGURE 2:**
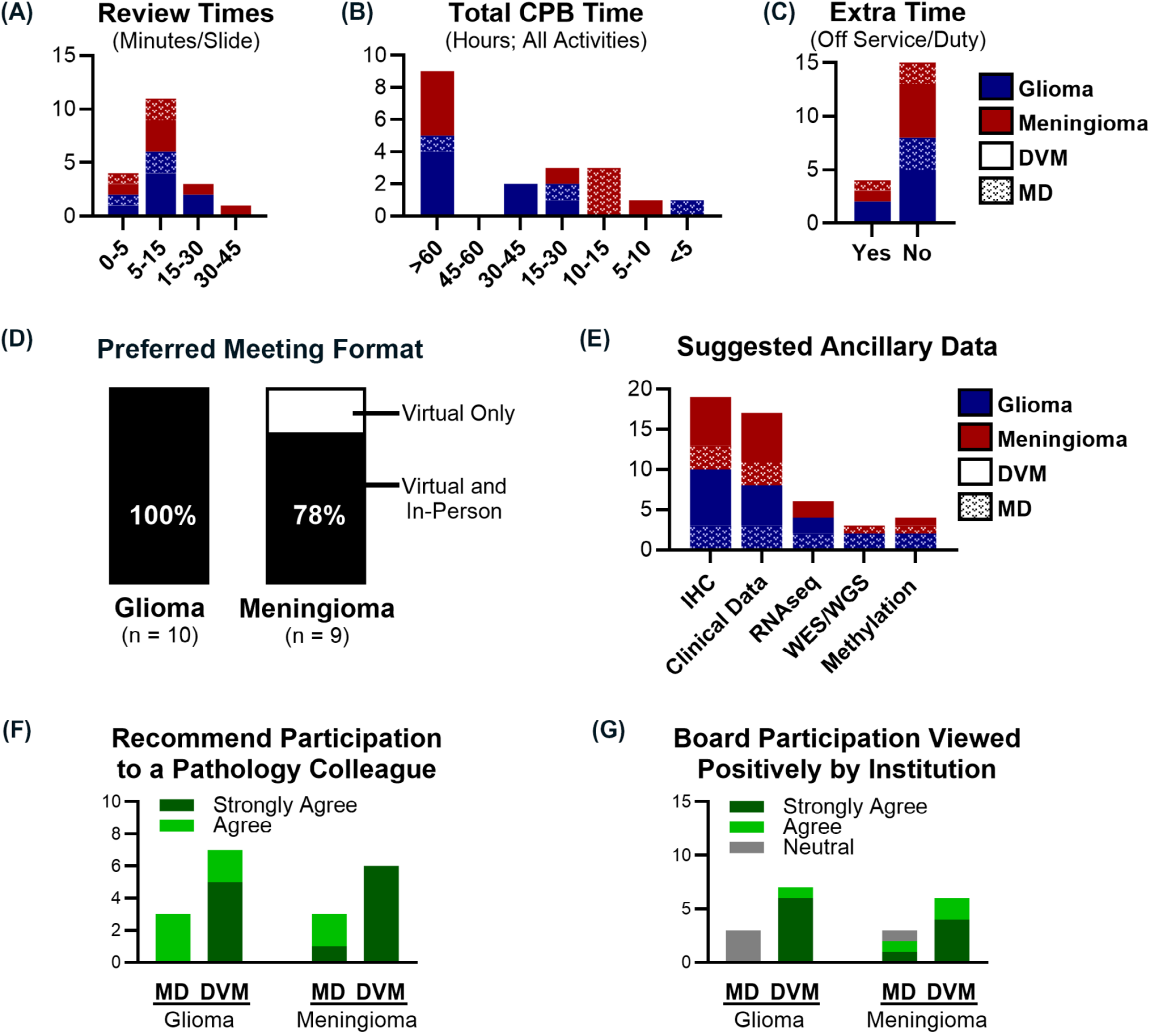
Feedback from surveyed pathologists for future comparative pathology boards (CPBs). (A) Average review time (minutes/slide) reported by number of survey respondents. (B) Total amount of time spent in support of the CPB reported by number of survey respondents. (C) Number of respondents that reported receiving time off service or duty to support their CPB participation. (D) Percent of CPB members that preferred a combination of virtual and in‐person meetings. (E) Number of CPB members that suggested each of the indicated data as a minimum requirement for case inclusion in future CPBs. (F) Number of respondents that would strongly agree, agree, or neither agree nor disagree that their CPB participation was viewed positively by their institution. (G) Number of respondents that agree or strongly agree that they would recommend participation on a CPB to a colleague.

Although the COVID19 pandemic has greatly altered the collaborative science landscape with a push toward virtual meetings, most members preferred a combination of in‐person and virtual meetings (Figure 2D). In‐person meetings are an opportunity to discuss the cross‐species landscape of disease and outline goals which should be reiterated throughout the trajectory of the CPB. While many pathologists are familiar with individuals within their own specialty, an in‐person meeting allows formal introductions of members and their backgrounds and enables interactions between fields; this facilitates research collaborations beyond the CPB. The glioma CPB convened an in‐person kickoff meeting which was positively received by members as a welcomed opportunity to exchange ideas and experiences. All CPBs have utilized virtual meetings to discuss histopathology and update classification systems prior to case review. A post‐review in‐person meeting can also be useful for discussing disparate features or defining a CPB consensus diagnosis to evaluate against outcome or ancillary data.

Compiling clinical metadata in advance of the slide review helps shape the best dataset. The suggested minimum requirement includes clinical data and immunohistochemistry (Figure 2E). Retrospective studies may have cases with different treatments and incomplete clinical details or outcome measures, which affect the study's ability to assign prognostic value to histologic features. Canine clinical trials offer a more straightforward method to examine histology against outcome. Inclusion of additional molecular adjuncts is more likely to uncover canine subpopulations with significant relevance to human patients; examples include the identification of similar subgroups in human and canine meningiomas through methylation profiling and RNA sequencing [6, 7].

All survey takers agreed or strongly agreed that they would recommend CPB participation to a colleague (Figure 2F). Most DVM‐trained survey participants agreed or strongly agreed that their institution viewed their participation on the CPB favorably (Figure 2G) and included it in promotion or performance evaluations. Several also requested letters of support from the COP. In contrast, most MD‐trained pathologists neither agreed nor disagreed, and none requested letters of support or specifically mentioned the CPB in performance evaluations, suggesting that CPB participation was less significant to their career development. As such, it is important that efforts are made to ensure that MD‐trained pathologists benefit from their involvement, including ensuring that their views are incorporated and that CPB goals are sufficiently comparative in nature.

When animals are proposed as models for human diseases, there is value in convening a CPB to assess the underlying histology and define features shared with human patients. Future CPBs should prioritize diseases for which animal models are most needed. Although our work was initiated in brain tumors [1, 4], we have expanded to osteosarcoma, which is estimated to occur 10 times more frequently in canine compared to pediatric patients, underscoring its value as a model and biospecimen resource [8]. Other canine cancers reviewed by teams of MD‐ and DVM‐trained pathologists include melanoma [9], urothelial carcinoma [10], and soft tissue tumors [11]. Collecting feedback from additional pathologists from different institutions and in different disease contexts will be important for establishing the value of CPBs and for outlining goals for future studies in this space. These interdisciplinary efforts support the use of canine clinical trials and tissue biospecimens to further canine cancer research while enhancing the translational relevance of comparative oncology for human patients.

## AUTHOR CONTRIBUTIONS

All authors participated in the COP's CPBs. JAB wrote the initial manuscript draft. All authors reviewed and approved the final draft.

## FUNDING INFORMATION

This work was supported by the Intramural Program of the National Cancer Institute, NIH (Z01‐BC006161). The content of this publication does not necessarily reflect the views or policies of the Department of Health and Human Services, nor does mention of trade names, commercial products, or organizations imply endorsement by the US government.

## CONFLICT OF INTEREST STATEMENT

The authors declare that they have no competing interests.

## Supporting information


**Data S1.** Supporting Information.

## Data Availability

The data that support the findings of this study are available from the corresponding author upon reasonable request.
