# Supplementary material for: Comparative pathology boards facilitate the translation of knowledge between canine and human cancer patients
Source: Brain Pathol. 2025 May 5;35(5):e70013. doi: 10.1111/bpa.70013 (PMC12352920; doi:10.1111/bpa.70013)
Supplement: Supplementary file 1 — Data S1. Supporting Information. [file BPA-35-e70013-s001.pdf]

Supplemental Figure 1. Goals of comparative pathology boards (CPBs)

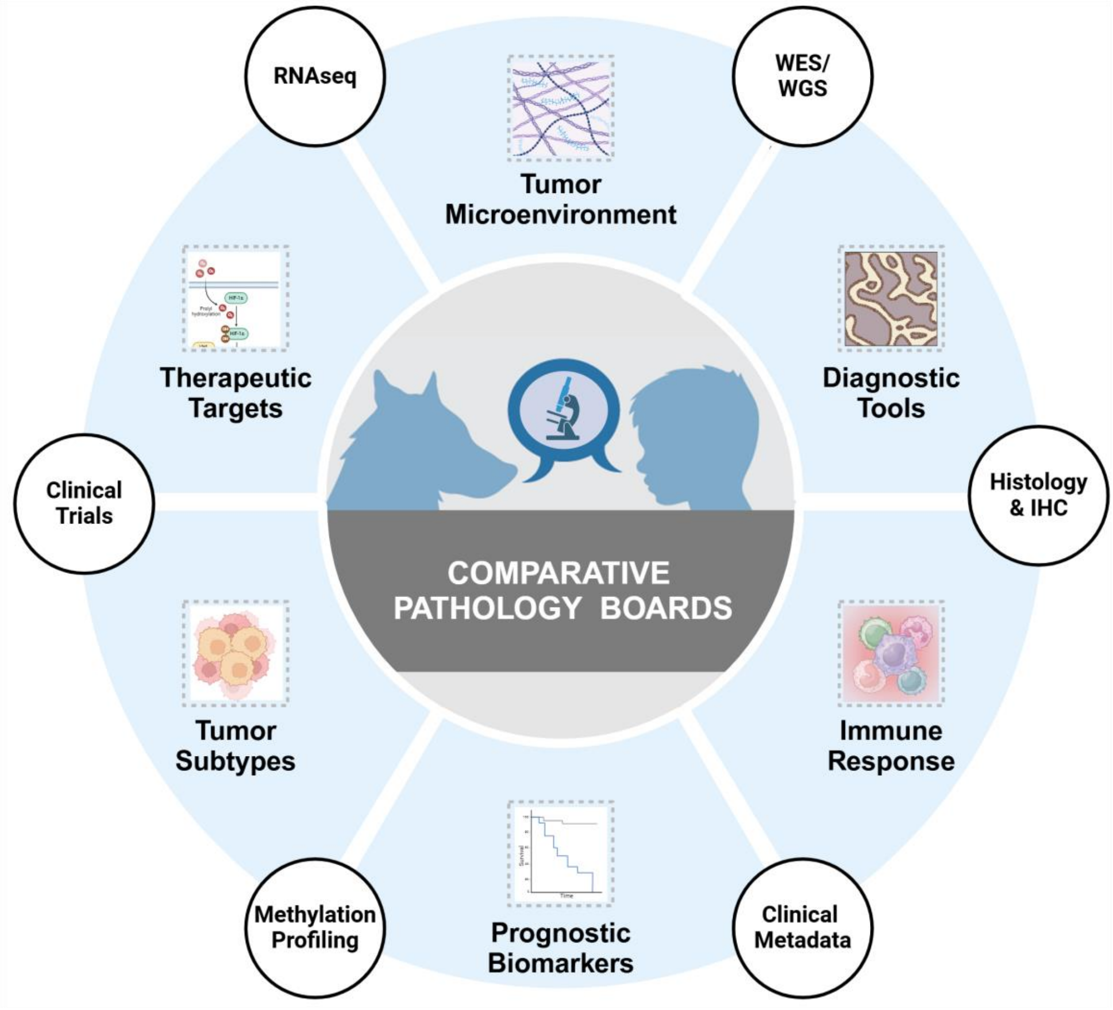

Supplemental Figure 1. Goals of comparative pathology boards (CPBs). CPB objectives may include reclassification of tumor subtypes, characterization of the tumor microenvironment, or identification of tissue-based biomarkers with prognostic, diagnostic, or therapeutic relevance. These efforts are supported by inclusion of ancillary data including sequencing, immunohistochemistry, methylation profiling, or clinical metadata.

Supplemental Figure 2. Composition of Surveyed CPB Members

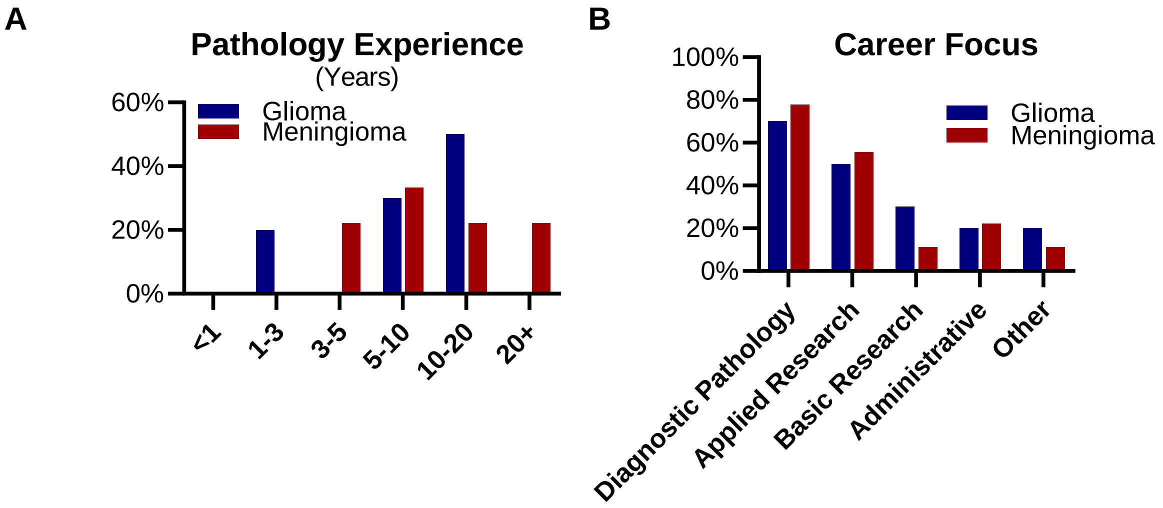

Supplemental Figure 2. Composition of surveyed comparative pathology board (CPB) members. (A) Reported years of pathology experience and (B) career focuses of the survey respondents including a combination of diagnostic pathology, research, and administrative duties.
